# Prone to food in bad mood—Emotion‐potentiated food‐cue reactivity in patients with binge‐eating disorder

**DOI:** 10.1002/eat.23683

**Published:** 2022-01-24

**Authors:** Ann‐Kathrin Arend, Rebekka Schnepper, Annika Petra Christine Lutz, Katharina Naomi Eichin, Jens Blechert

**Affiliations:** ^1^ Division of Health Psychology, Department of Psychology Paris‐Lodron‐University of Salzburg Salzburg Austria; ^2^ Centre for Cognitive Neuroscience, Paris‐Lodron‐University of Salzburg Salzburg Austria; ^3^ Department of Behavioural and Cognitive Sciences, Institute for Health and Behaviour University of Luxembourg Esch‐sur‐Alzette Luxembourg

**Keywords:** binge‐eating disorder, corrugator supercilii, desire to eat, electromyography, emotion induction, emotional eating, food‐cue reactivity, pleasantness

## Abstract

**Objective:**

Theories on emotional eating are central to our understanding of etiology, maintenance, and treatment of binge eating. Yet, findings on eating changes under induced negative emotions in binge‐eating disorder (BED) are equivocal. Thus, we studied whether food‐cue reactivity is potentiated under negative emotions in BED, which would point toward a causal role of emotional eating in this disorder.

**Methods:**

Patients with BED (*n* = 24) and a control group without eating disorders (CG; *n* = 69) completed a food picture reactivity task after induction of negative versus neutral emotions. Food‐cue reactivity (self‐reported food pleasantness, desire to eat [DTE], and corrugator supercilii muscle response, electromyogram [EMG]) was measured for low‐ and high‐caloric food pictures.

**Results:**

Patients with BED showed emotion‐potentiated food‐cue reactivity compared to controls: Pleasantness and DTE ratings and EMG response were increased in BED during negative emotions. This was independent of caloric content of the images.

**Conclusions:**

Food‐cue reactivity in BED was consistent with emotional eating theories and points to a heightened response to all foods regardless of calorie content. The discrepancy of appetitive ratings with the aversive corrugator response points to ambivalent food responses under negative emotions in individuals with BED.

## 1 INTRODUCTION

Eating for reasons other than hunger is common in healthy individuals despite the fact that it contributes to an unfavorable nutritional profile and ill health (Guh et al., 2009; Tuthill, Slawik, O'Rahilly, & Finer, 2006). One popular explanation for such nonhomeostatic food intake is *emotional eating* in response to negative emotions as intake of palatable foods can reduce negative emotions (Macht & Simons, 2011; van Strien, Gibson, Baños, Cebolla, & Winkens, 2019). Emotional eating theories are particularly relevant to eating disorders such as bulimia nervosa (BN) or binge‐eating disorder (BED), as they predict when the occurrence of binge eating is more likely and explain its maintenance through negative reinforcement (Macht, 2008; Macht & Simons, 2011). Hence, it is surprising that recent meta‐analyses (Cardi, Leppanen, & Treasure, 2015; Evers, Dingemans, Junghans, & Boevé, 2018) had discrepant findings and called the basic phenomena of emotion‐potentiated eating and its role in BED into question. Thus, the present study set out to re‐examine the causal role of laboratory‐induced negative emotions for appetitive responding in BED.

Most of the laboratory‐based research on emotional eating focuses on food intake measures as a dependent variable (Cardi et al., 2015; Evers et al., 2018). While actual food intake has high external validity, *food‐cue reactivity* paradigms (measuring experiential and psychophysiological responses to food cues) might have advantages, as they tap into the same underlying appetitive tendencies but might be less regulated than overt eating. Food‐cue reactivity measures are validated through relationships with excessive food intake, binge eating, and weight gain (Boswell & Kober, 2016; Jansen, 1998; Nederkoorn & Jansen, 2002; Wardle, 1990).

One promising index of food‐cue reactivity—aside from ratings on appetitive responses—is facial electromyogram (EMG) of the corrugator supercilii (“frown”) muscle, as EMG has been shown to be sensitive to food cues, emotional context manipulations and binge‐eating symptomatology (e.g., Jackson, Malmstadt, Larson, & Davidson, 2000; Schnepper et al., 2020, 2021; Svaldi, Tuschen‐Caffier, Peyk, & Blechert, 2010). Leehr et al. (2016) and Svaldi et al. (2010) documented increased appetitive ratings of food images in BED alongside increased (aversive) EMG responses. This points to an ambivalent response pattern, where EMG seems to tap into the negative and potentially more implicit components of the response. In contrast to those findings, Schnepper et al. (2021) reported *de*creased (appetitive) EMG responses to high‐calorie food cues in BN, during negative compared to neutral emotions, in a task that was identical to the present one (mood induction based on idiosyncratic scripts and repeated assessments of different food‐cue reactivity measures after presentation of food and object pictures). A further cue‐reactivity study in BED found impaired inhibitory control (antisaccade task) and a decrease of conflict‐related electroencephalography‐indices during a task requiring disengaging attention from food stimuli during negative emotions (no neutral condition; Leehr et al., 2018).

On the background of the high relevance of the emotional eating theory for treatment of BED and understanding of BED etiology, the present study aimed to follow‐up on discrepant findings regarding emotion‐potentiated food intake by shedding light on the causal role of emotions for appetitive responding. Thus, patients with BED and controls (“Group”) underwent neutral and negative emotion inductions (“Condition”) while high‐ and low‐caloric food pictures (“Calories”) and object pictures were presented. We expected three‐way interactions (“Group*Content*Calories”) with elevated *appetitive* pleasantness and desire to eat (DTE) ratings in BED during negative emotions for high‐calorie foods. The literature does not allow a directed hypothesis for corrugator reactivity as both *aversive* (heightened EMG; Leehr et al., 2016; Svaldi et al., 2010) and *appetitive* (attenuated EMG in BN; Schnepper et al., 2021) responses have been described.

## 2 METHOD

### 2.1 Participants

Participants of female sex, with BED (*n* = 24) or without lifetime eating disorders (control group [CG]; *n* = 65) were tested at three sites (see Table S3). Two interviews (German versions of the Eating Disorder Examination and the Structured Clinical Interview for DSM‐IV; Hilbert & Tuschen‐Caffier, 2016; Wittchen, Zaudig, & Fydrich, 1997) were combined to allow diagnoses according to DSM‐5. Exclusion criteria were current substance abuse, psychotic or neurological disorders, vegetarianism, veganism, diabetes, pregnancy and skin or food allergies.

### 2.2 Procedure

#### 2.2.1 General procedure

Laboratory sessions were scheduled at ~3 p.m. and participants were instructed to consume standardized lunch options (~550 kcal) 3 h before. Participants completed informed consent, and records of food intake, emotional state, and current hunger. An interview for idiosyncratic, script‐based emotion induction inquired about recent intense, negative emotional situations (e.g., Blechert, Goltsche, Herbert, & Wilhelm, 2014) out of which the most vivid and negative (nontraumatic) situation was chosen and condensed into eight stimulus sentences to be shown during the task. A standardized situation (either brushing teeth or going to work/university/school/shopping) generated sentence for the neutral condition. Several physiological sensors were then attached (~20–40 min), and after an interoception task (~10 min) the food‐cue reactivity task took place.

**FIGURE 1:**
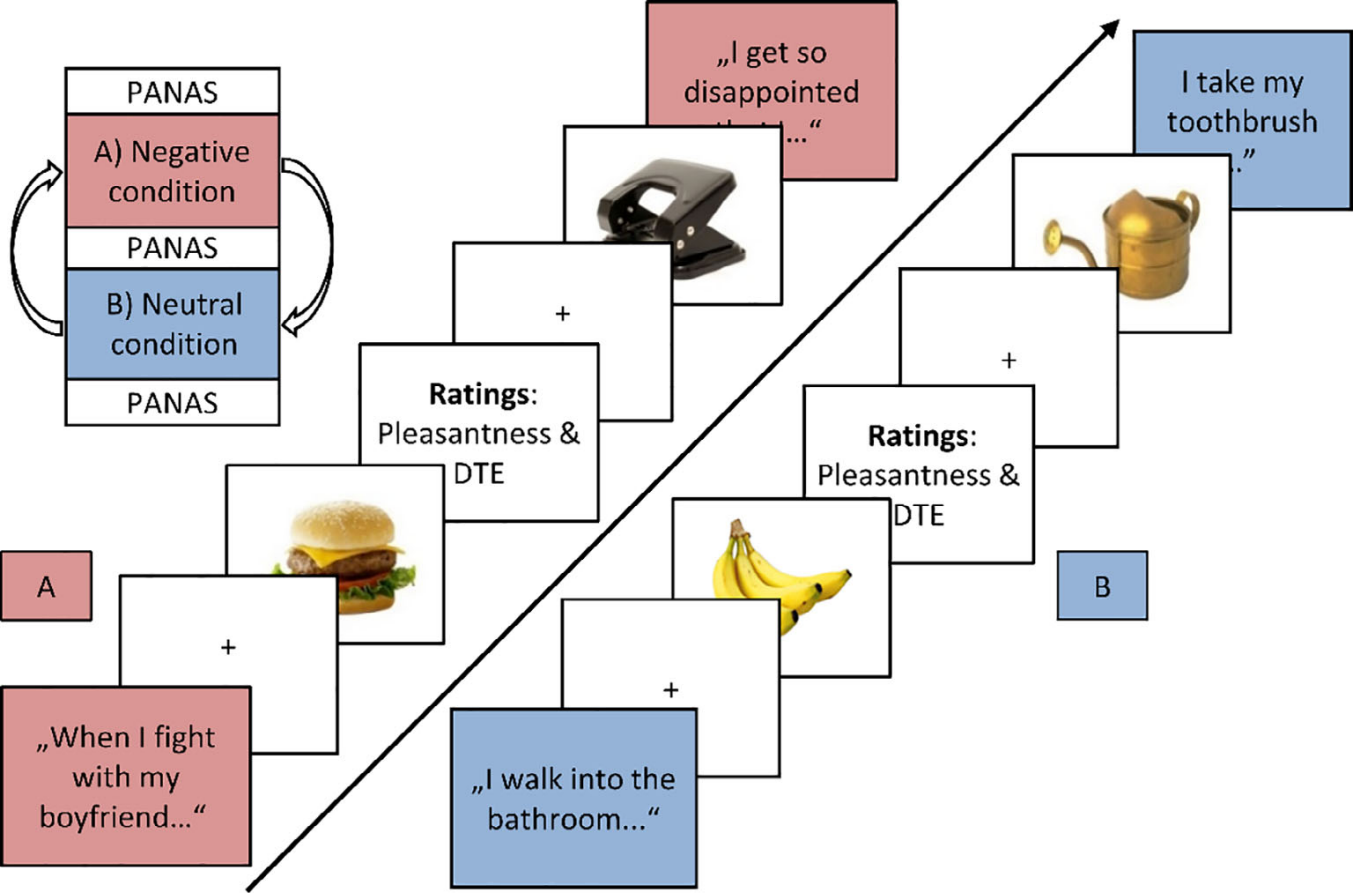
Exemplary on‐screen trial sequence of the (a) negative and the (b) neutral condition. Sentences from the idiosyncratic script are interleaved with presentations of food/object images. These images are preceded by a fixation cross and followed by respective ratings (pleasantness and desire to eat [DTE]). The order of conditions is randomized between participants and after each condition a questionnaire assessed emotional state (Positive and Negative Affect Schedule; PANAS)

After a relaxation phase (~1 min), the corresponding sentences for each condition (neutral and negative, in counterbalanced order across participants) were read to the participant and then presented on‐screen, interleaved between food and object pictures, and picture‐rating prompts (see Figure 1). Object and food pictures, 26 each (high‐ and low‐caloric, 13 each; see Table S16; Blechert, Lender, Polk, Busch, & Ohla, 2019) were presented twice per condition in randomized order but rated only once (randomized at the first or second presentation). A ~5‐min “washout” phase separated the conditions.

### 2.3 Measures

*Negative emotions* were measured averaging the 10 negative affective items of the Positive and Negative Affect Schedule (PANAS state; Krohne, Egloff, Kohlmann, & Tausch, 1996) at baseline, after the negative and the neutral condition (Cronbach's *α* negative subscales ≥.859).

*Pleasantness and DTE* were rated on visual analog scales (0–100) for food pictures (pleasantness also for objects). A pleasantness difference score was calculated ((individual food picture rating) − (mean of object picture ratings, within condition, within participant)) to control for nonfood specific reactivity.

*EMG* reactivity to the images (at every picture presentation) was recorded with miniature Ag/AgCl electrodes over the left corrugator supercilii muscle, following Fridlund and Cacioppo (1986). After high pass (28 Hz) and notch (50 Hz) filtering, rectification and smoothing (50 ms moving average), manual artifact inspection was done. Seven participants (BED *n* = 4, CG *n* = 3) were excluded from EMG analyses due to poor signal quality. From mean signal amplitude (0–2,500 ms) a mean prestimulus baseline (−500–0 ms) was subtracted. To reduce the impact of overly influential cases, first, EMG scores were winsorized (2.5th and 97.5th percentile) within participant, and second, remaining influential cases (with values >4*mean cook's distance) were removed (BED *n* = 2, CG *n* = 3). Nonfood specific EMG reactivity was controlled by subtracting mean object reactivity.

### 2.4 Statistical analyses

Linear mixed‐effect models (LMMs) were used to model the full variance on the trial level (Nezlek, 2008). The models included a random intercept for “Participants”, a random slope for “Calories” and the fixed factors “Condition*Calories*Group” (see Analysis S2 for specifications). Post hoc tests were calculated for significant interactions including “Group.”

## 3 RESULTS

### 3.1 Participants

The BED group did not differ significantly from the CG in age, body mass index (BMI) and years of education (all *p*'s ≥ .102). Self‐reported trait emotional eating and external eating, baseline hunger, hunger after the task, eating behavior pathology, anxiety, depressive symptoms, and impulsivity were significantly higher in patients with BED compared to CG (all *p*'s ≤ .049, see Table S1).

### 3.2 Emotion manipulation check

PANAS scores indicated generally more negative emotions in the BED group, yet both groups showed comparable and significant increases in PANAS scores in the negative compared to the neutral condition, affirming successful emotion induction in both groups (see Analysis S1).

### 3.3 Food‐cue reactivity

#### 3.3.1 Pleasantness and DTE

**FIGURE 2:**
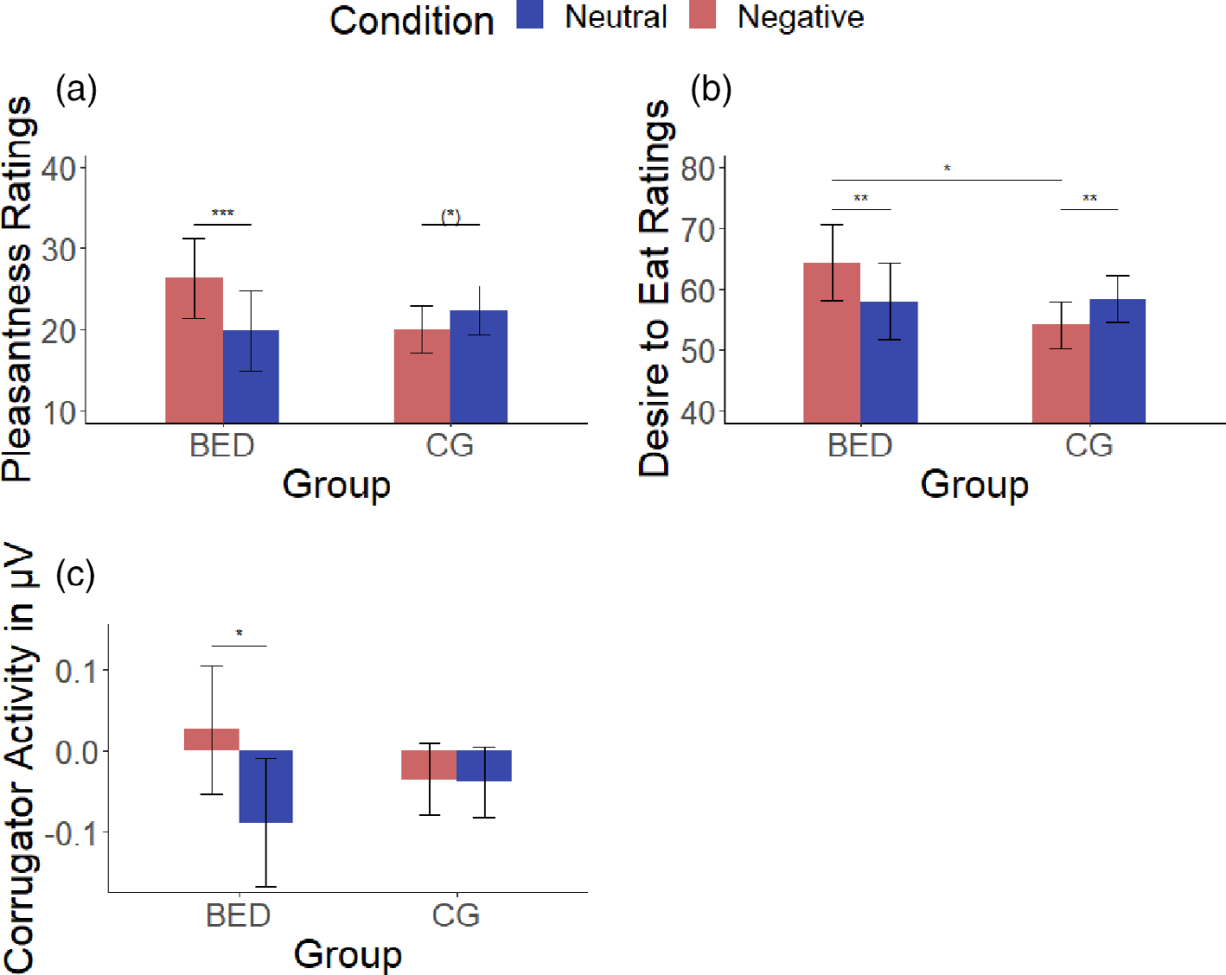
Group means in the neutral and negative condition for pleasantness, DTE, and corrugator. (a) Pleasantness ratings of foods—objects difference scores, (b) desire to eat ratings of food pictures, and (c) relative corrugator activity. Corrugator values are baseline corrected, food − object difference scores. Error bars represent the 95% confidence interval around the mean. Control group (CG); patients with binge‐eating disorder (BED). (a and b): CG *n* = 65, BED *n* = 24; (c): CG *n* = 60, BED *n* = 18. See Figures S3 and S4, which cover the calorie factor. Significance codes for post hoc tests of the significant Group*Condition interactions are indicated as: ***p < .001; **p < .010; *p < .050; ^(^*^)^ *p* < .100. DTE, desire to eat

On both rating scales, main effects of condition (pleasantness: *β* = 5.00, *SE* = 1.32, *t*(4550) = 3.78, *p* < .001 / DTE: *β* = 6.95, *SE* = 1.54, *t*(4550) = 4.52, *p* < .001) were found, each modulated by Condition*Group interactions (pleasantness: *β* = −8.96, *SE* = 2.48, *t*(4550) = −3.62, *p* < .001 / DTE: *β* = −11.64, *SE* = 2.87, *t*(4550) = −4.05, *p* < .001). BED patients reported higher pleasantness / DTE in the negative compared to the neutral condition (post hoc tests: *t*(4550) = 6.46, *p* < .001 / *t*(4550) = 3.72, *p* = .001). CG participants tended to show the reversed pattern (post hoc tests: *t*(4550) = −2.38, *p* = .054 / *t*(4550) = −3.86, *p* = .001). During the negative condition BED reported higher DTE compared to CG (post hoc test: *t*(101) = −10.16, *p* = .038; see Figure 2a,b).

Furthermore, Condition*Calorie interactions (pleasantness: *β* = −5.25, *SE* = 1.87, *t*(4550) = −2.81, *p* = .005 / DTE: *β* = −5.51, *SE* = 2.17, *t*(4550) = −2.54, *p* = .011) were found, but were independent of group (no three‐way interactions: *β* = 0.25, *SE* = 3.50, *t*(4550) = 0.07, *p* = .942 / *β* = 2.12, *SE* = 4.06, *t*(4550) = 0.52, *p* = .601) and thus not further followed (see Figure S3A,B). No other effects/interactions reached significance (all *p*'s ≥ .209 / *p*'s ≥ .104). Both models had medium effect sizes (*conditional pseudo R* ^2^ = 0.21/0.23).

#### 3.3.2 Electromyogram

A Condition*Group interaction (*β* = 0.12, *SE* = 0.06, *t*(7873) = 2.14, *p* = .033) indicated a relative frowning reaction in BED patients when viewing food in the negative condition compared to the neutral condition (post hoc test: *t*(7867) = −0.11, *p =* .005; see Figure 2c) which was not seen for CG participants (post hoc test *t*(7865) = 0.00, *p =* .998).

Neither the three‐way interaction (*β* = −0.02, *SE* = 0.08, *t*(7873) = −0.20, *p* = .840), nor any other calorie effects were significant (all other *p*'s ≥ .278). The model had a small effect size (*conditional pseudo R* ^2^ = 0.04).

Significance levels of the Group*Condition interactions (for pleasantness, DTE, and EMG) did not change after controlling for study site, BMI or compliance with standardized lunch options (see Tables S9–S11). Also, see supplements for detailed model specifications, all predictor values and post hoc tests (Tables S7 and S8).

## 4 DISCUSSION

The present study is the first to investigate emotion‐potentiated food‐cue reactivity in BED. Our idiosyncratic, script‐based emotion induction was successful and led to a similar increase of negative emotions in both groups. In line with our hypothesis, individuals with BED showed increased pleasantness and DTE ratings in the negative condition compared to the neutral, while controls tended to show the reversed pattern. This *appetitive* pattern is contrasted with increased EMG activity and thus an *aversive‐defensive* physiological response in the negative condition in BED. Contrary to our hypothesis, none of this was specific to pictures with high‐calorie content.

The results on self‐report ratings support the causal role of negative emotions for appetitive of food‐cue reactivity in BED (Cardi et al., 2015; Leehr et al., 2018). This is a well‐validated proxy for food intake and weight gain and might thus predispose patients to binge eating in certain instances (Boswell & Kober, 2016; Jansen, 1998; Nederkoorn & Jansen, 2002; Wardle, 1990). As such, our findings contrast with work that called the role of emotional eating (in BED compared to CG) into question (Evers et al., 2018)—at least in regard to self‐reported food‐cue reactivity.

Responses of corrugator (“frown muscle”), pointed to an interesting discrepancy: internal conflict when exposed to food cues—possibly related to a loss of control threat. This also contrasts with findings of appetitive EMG responses in BN (Schnepper et al., 2021). BN might differ from BED since patients might anticipate compensations after binging and thus, experience less threat of the consequences. Similarly, we found emotion‐potentiated food‐cue reactivity in BED for both high‐ and low‐calorie pictures, whereas BN patients showed this pattern only for high‐calorie foods (Lutz et al., 2021; Schnepper et al., 2021). This generalized response pattern might relate to the present sample with elevated BMIs in both BED and CG and potentially higher intake of a broad range of foods. Future studies should examine the role of BMI in emotion‐potentiated food‐cue reactivity as a function of calorie density.

The study had various strengths in design and analysis (e.g., highly controlled laboratory setting, a large, CG and use of LMMs), which support internal validity of the findings. Yet, higher negative reactivity in BED (Leehr et al., 2015; Lingswiler, Crowther, & Stephens, 1987)—despite comparable emotion induction strength in both groups (see Analysis S1)—could be addressed by adding a clinical CG with depressive symptomatic and thus similarly high negative emotional reactivity. Furthermore, although food‐cue reactivity has clear advantages, it should be backed up by food intake measures in future studies to maximized external validity. Clearly, the patient sample size should be increased, as the current study is underpowered with regard to findings in EMG and the nonsignificant three‐way interaction (see Analysis S2, S3).

To conclude, the present findings are consistent with theories such as the emotion‐regulation model of binge eating (Leehr et al., 2015). They also have therapeutic implications: Food‐specific inhibitory trainings (i.e., antisaccade trainings; Giel, Speer, Schag, Leehr, & Zipfel, 2017; Schag et al., 2019) should be most effective under negative emotions. Finally, the present results back up the indication of emotion‐regulation trainings in binge eating.

## CONFLICT OF INTERESTS

The authors have no conflict to declare.

## Supporting information


**Appendix** S1. Supporting Information

## Data Availability

Data and analyses codes are available at the Open Science Framework https://doi.org/10.17605/OSF.IO/Y9B2Z.
