# Supplementary material for: Prone to food in bad mood—Emotion‐potentiated food‐cue reactivity in patients with binge‐eating disorder
Source: Int J Eat Disord. 2022 Jan 24;55(4):564–9. doi: 10.1002/eat.23683 (PMC9303400; doi:10.1002/eat.23683)
Supplement: Supplementary file 1 — Appendix S1. Supporting Information [file EAT-55-564-s001.docx]

**Supplements**

**Further Measures**

**Body-Mass-Index.** BMI (kg/m²) was calculated from height and weight, which were measured at the end of each laboratory session.

**Hunger.** Participants rated four items regarding hunger, food craving and fullness on nine-point Likert-scales: ‘How hungry are you right now?’; ‘How strong is you craving for food right now?’; ‘What do you think how much food you could eat right now?’ and ‘How full feels your stomach right now?’ (1=’not at all/very weakly’, to 9=’never been this hungry/very strongly/huge amounts/very full’). They rated these at baseline (before the experiment started) and after the emotional food-cue reactivity task. The last item was inverted and sum scores from these four items were calculated for both time points.

**Imagination.** After the negative and after the neutral condition of the food-cue reactivity task, participants rated the item ‘How well were you able to imagine the situation?’ on a nine-point Likert-scale (1=’I thought of the situation but could not imagine it.’, to 9=’I imagined the situation as clearly as if it was real.’).

**Eating Disorder Pathology.** Three subscales of the Eating Disorder Inventory 2 (EDI-2; Paul & Thiel, 2005) were used to assess eating disorder pathology: ‘body dissatisfaction’ (items: 2, 9, 12i, 19i, 31i, 45, 55i, 59, 62i), ‘bulimia’ (items: 4, 5, 28, 38, 46, 53, 61) and ‘drive for thinness’ (items: 1i, 7, 11, 16, 25, 32, 49). Participants rated the items on a six-point Likert-scale (1=’never’ to 6=’always’). Items marked with an ‘i’ were inverted. Finally, all items were aggregated to a sum score for each of the three subscales.

**Research Diagnoses.** Research diagnoses of binge-eating disorder (BED), depressive, anxiety, obsessive-compulsive and post-traumatic stress disorders were conducted with the Structured Diagnostic Interview for DSM-IV (German version; Wittchen et al., 1997) and the Eating Disorder Examination (EDE; German version: Hilbert & Tuschen-Caffier, 2006).

**Binges.** The number of objective binge-eating episodes (with objective overeating and loss of control eating) over the last three months before the interview was assessed with the EDE (German version: Hilbert et al., 2004).

**Trait Emotional Eating.** The Salzburg Emotional Eating Scale (SEES; Meule et al., 2018) was used to measure dispositional tendencies of less, unchanged or increased food consumption in reaction to happiness, sadness, anger, anxiety or overall negative emotions. The subscales for happiness, sadness, anger and anxiety were measured with 5 items each. These items were rated on a five-point Likert-scale (1=’I eat much less than usual’ to 3=’I eat just as much as usual’ to 5=’I eat much more than usual’). Mean scores were calculated for all four subscales. The overall negative subscale comprises a mean score of all 15 items from the sadness, anger and anxiety subscales.

Also, the emotional eating subscale of the question version (33 items) of the Dutch Eating Behavior Questionnaire (DEBQ; van Strien et al., 1986) was used to measure the dispositional tendency to overeat in reaction to negative emotion. The items were rated on a five-point Likert-scale (1=’never’ to 5=’very often’). A mean score was calculated from the corresponding items (11, 12, 13, 14, 15, 16, 17, 18, 19, 20, 21, 22, and 23).

**Trait Restrained Eating.** The Restrained Eating subscale of the DEBQ (van Strien et al., 1986) was used to measure the dispositional tendency of trying to restrain ones food intake. The items were rated on a five-point Likert-scale (1=’never’ to 5=’very often’). A mean score was calculated from the corresponding items (1, 2, 3, 4, 5, 6, 7, 8, 9, and 10).

**Trait External Eating.** The External Eating subscale of the DEBQ (van Strien et al., 1986) was used to measure the dispositional tendency to overeat in reaction to external food cues. The items were rated on a five-point Likert-scale (1=’never’ to 5=’very often’). Item 31 was inverted and a mean score was calculated from the corresponding items (24, 25, 26, 27, 28, 29, 30, 31i, 32, and 33).

**Trait Impulsiveness.** The short form of the Barratt Impulsiveness Scale (BIS-15; Meule et al., 2011) was used to measure dispositional impulsivity. The 15 items were rated on a four-point Likert-scale (1=’ rarely/never’ to 4=’very often/always’). The items 1i, 4i, 5i, 7i, 8i, and 15i were inverted and a sum score was calculated.

**Depression.** Depressive symptoms were assessed with the short version of the Center for Epidemiologic Studies - Depression Scale (CESD; Radloff, 1977). The 15 items were rated on a four-point Likert-scale (0=’rarely or never (less than one day)’ to 3=’most of the time, always (for 5 to 7 days)’). The items 9i, and 12i were inverted and a sum score was calculated.

**Trait Anxiety. The Trait Subscale of the State and Trait Anxiety Inventory** (STAI-T; Grimm, 2009; Spielberger, 1983) was used to measure dispositional anxiety. The 20 items of the STAI-T were rated on a four-point Likert-scale (1=’almost never’ to 4=’almost always’). The items 1i, 6i, 7i, 10i, 13i, 16i, and 19i were inverted and a sum score was calculated.

**Compliance with Lunch Instructions.** Starting the laboratory session participants filled in a food record for the day. They were asked ‘if’ they ate something for breakfast and lunch at the day of the laboratory session. If they answered ‘yes’ they were further asked ‘what’ and ‘how much’ they ate. Lastly, they had to answer ‘when’ they ate their last main meal. This information from each participant was used to control compliance with lunch time instructions. Beforehand, participants were instructed to choose from and consume one of several lunch options standardized in calorie content (~550 kcal each)~3 hours before the laboratory session. The compliance with these instructions was coded as 0=‘compliant’, 1=‘non-compliant (ate less/nothing)’, 2=‘non-compliant (ate more)’ for each participant.

**Descriptive Statistics and Group Differences**

**Table-S1**

Means, standard deviations, ranges, t-tests and percentages for group differences on descriptive measures

|  | BED (*n*=24) | CG (*n*=65) |  |
| --- | --- | --- | --- |
| **Variable** | ***M* (*SD*, range)** | ***M* (*SD*, range)** | **statistic** |
| Age (in years) | 33.3 (11.1, 18-52) | 29.9 (8.4, 21-56) | *t*(87) = -1.39, *p* = .174 |
| BMI (kg/m²) | 30.1 (5.9, 21.2-43.9) | 27.9 (5.3, 20.2 – 42.1) | *t*(87) = -1.66, *p* = .102 |
| Years of education | 16.0 (4.1, 8-25) | 16.1 (2.7, 9-22) | *t*(87) = 0.16, *p* = .874 |
| Hunger baseline | 20.6 (6.3, 4-32) | 17.7 (6.0, 7-36) | *t*(87) = -2.00, *p* = .049 |
| Hunger post task | 27.5 (5.0, 15-35) | 24.5 (5.7, 8-36) | *t*(87) = -2.91, *p* = .005 |
| Imagination negative condition | 7.1 (1.6, 3-9) | 6.9 (1.8, 1-9) | *t*(87) = -0.35, *p* = .729 |
| Imagination neutral condition | 6.3 (1.8, 2-9) | 6.7 (1.8, 2-9) | *t*(86) = 0.90, *p* = .711 |
| EDI-2-body dissatisfaction | 42.4 (6.9, 28-53) | 36.6 (10.5, 16-54) | *t*(86) = -2.99, *p* = .004 |
| EDI-2-bulimia | 25.4 (7.1, 13-38) | 15.5 (7.6, 7-41) | *t*(86) = -5.51, *p* < .001 |
| EDI-2-drive for thinness | 29.3 (7.5, 16-42) | 23.8 (9.1, 8-41) | *t*(86) = -2.59, *p* = .011 |
| Binges (last three months) | 36.6 (32.7, 12-153) | - |  |
| SEES_negative_ | 3.8 (0.7, 2.5-5.0) | 3.0 (0.5, 1.8-4.5) | *t*(87) = -5.78, *p* < .001 |
| SEES_sadness_ | 4.2 (0.6, 3.0-5.0) | 3.5 (0.6, 2.2-4.8) | *t*(87) = -4.84, *p* < .001 |
| SEES_anger_ | 3.8 (0.9, 2.2-5.0) | 2.8 (0.8, 1.0-4.2) | *t*(87) = -4.88, *p* < .001 |
| SEES_anxiety_ | 3.5 (1.1, 1.0-5.0) | 2.8 (0.7, 1.0-4.6) | *t*(87) = -2.95, *p* = .006 |
| SEES_happiness_ | 2.5 (0.8, 1.4-4.4) | 2.8 (0.6, 1.0-4.0) | *t*(87) = 1.59, *p =* .117 |
| DEBQ_emotional_ | 3.9 (0.6, 2.4-5.0) | 2.69 (0.8, 2.3-4.5) | *t*(87) = -6.78, *p* < .001 |
| DEBQ_restrained_ | 3.05 (0.7, 2.8-5.0) | 2.75 (0.6, 1.0-4.8) | *t*(87) = -1.92, *p =* .058 |
| DEBQ_external_ | 3.68 (0.6, 1.9-4.2) | 3.38 (0.6, 1.2-4.0) | *t*(87) = -2.22, *p =* .029 |
| Impulsiveness (BIS-15) | 36.88 (8.7, 21-51) | 32.37 (7.5, 20-55) | *t*(87) = -2.40, *p =* .019 |
| Depression (CESD) | 16.3 (10.9, 2-35) | 9.5 (6.8, 0-29) | *t*(87) = -3.05, *p* = .005 |
| Anxiety (STAI) | 49.4 (12.9, 28-71) | 39.9 (10.5, 22-60) | *t*(87) = -3.55, *p* < .001 |
|  | **BED (*n*=24)** | **CG (*n*=54)**† |  |
| **Variable** | **Percentage** | **Percentage** |  |
| Depressive disorders | 29 % | 4 % |  |
| Anxiety disorders | 54 % | 24 % |  |
| Obsessive-compulsive disorder | 8 % | 0 % |  |
| Post-traumatic stress disorder | 8 % | 0 % |  |
| Borderline personality disorder | 17 % | 2 % |  |

*Note*. Control group (CG) and binge-eating disorder (BED). Body-Mass-Index (BMI). Eating pathology (Eating Disorder Inventory 2, EDI-2, Paul & Thiel, 2005). Emotional eating (Salzburg Emotional Eating Scale, SEES, Meule et al., 2018) scores under three indicate less food intake in reaction to certain emotions, three indicates no change in food intake and scores over three indicate increased food intake in reaction to certain emotions. Dutch Eating Behavior Questionnaire (DEBQ; van Strien et al., 1986) subscales for emotional, restrained and external eating. Impulsiveness (short form of the Barrett Impulsiveness Scale, BIS-15; Meule et al., 2011). Depression (Center for Epidemiologic Studies - Depression Scale, CESD, Radloff, 1977). Trait anxiety (State and Trait Anxiety Inventory, STAI, Grimm, 2009; Spielberger, 1983). In case of variance inhomogeneity, values were adjusted but nominal *df* are reported for comprehensibility. Research diagnoses of psychological disorders were conducted with the structured diagnostic interview for DSM-IV (German version; Wittchen et al., 1997) and the eating disorder examination (German version: Hilbert & Tuschen-Caffier, 2006). †The percentages for current mental disorders in the CG were calculated for participants tested in Salzburg only (*n*=54) as CG participants tested in Luxemburg were excluded in case of any current mental disorders.

**Table-S2**

Frequencies and Chi-square results for group differences in ‘compliance to the instructed standardized lunch options’, ‘content of neutral condition’, ‘content of negative condition’ and ‘presentation order of conditions’ (*N*=89)

|  |  | *n* | % | *n* | % | χ²(2) | *p* |
| --- | --- | --- | --- | --- | --- | --- | --- |
| **Variables** | **Category** | **BED (*n*=24)** | | **CG (*n*=65)** | |  |  |
| **Lunch instruction** | Compliant | 21 | 87.50 | 64 | 98.46 | 5.3 | .070 |
|  | Non-compliant (less) | 1 | 4.17 | 0 | 0.00 |  |  |
|  | Non-compliant (more) | 2 | 8.33 | 1 | 1.54 |  |  |
|  |  |  |  |  |  |  |  |
| **Neutral condition content** | Brushing teeth | 21 | 87.50 | 50 | 78.13 | 1.2 | .270 |
|  | Going to work/school/ university/shopping | 3 | 12.50 | 15 | 23.08 |  |  |
|  |  |  |  |  |  |  |  |
| **Negative condition content** | Conflicts with partners | 5 | 4.17 | 8 | 12.31 | 13.0 | .073 |
|  | Conflicts with family members | 6 | 25.00 | 15 | 23.08 |  |  |
|  | Conflicts with friends | 0 | 0.00 | 12 | 18.46 |  |  |
|  | Conflicts with work related contacts | 4 | 16.67 | 13 | 20.00 |  |  |
|  | Other interpersonal situations | 6 | 25.00 | 6 | 9.23 |  |  |
|  | Non-social work situations | 2 | 8.33 | 7 | 10.77 |  |  |
|  | Body issues | 1 | 4.17 | 0 | 0.00 |  |  |
|  | Non-social other situations | 0 | 0.00 | 4 | 6.15 |  |  |
|  |  |  |  |  |  |  |  |
| **Presentation order of conditions** | Negative first | 13 | 54.17 | 26 | 40.00 | 1.4 | .232 |
|  | Neutral first | 11 | 45.83 | 39 | 60.00 |  |  |

*Note*. Control group (CG) and binge-eating disorder (BED).

**Table-S3**

Distribution of participants to the respective study sites.

| Group | Salzburg (Austria) | Luxembourg | Prien (Germany) |
| --- | --- | --- | --- |
| BED | 21 | 1 | 2 |
| CG | 54 | 11 | 0 |

*Notes.* Control group (CG) and patients with binge-eating disorder (BED).

**Analysis-S1**

**Emotion manipulation check and content of idiosyncratic scripts.**

Most of the situations for the negative condition involved conflicts with either family members (BED: 25% / CG: 23%), work related contacts (17% / 20%), friends (0% / 20%) and partners (4% / 12%). The rest chose other interpersonal situations (25% / 9%), various non-social situations (8% / 17%) and situations related to body issues (4% / 0%). There were no significant association of content in the negative condition and group, χ^2^(1, *N*=86)=13.0, *p*=.073. For the neutral condition participants either chose brushing their teeth (88% / 78%) or going to work/school/shopping by various means of transport (13% / 23%). Again, there was no significant association of content in the neutral condition and group, χ^2^(1, *N*=86)=1.2, *p*=.270 (See supplements, Table-S2).

Participants indicated that they could imagine the scenarios from the idiosyncratic scripts well (M=6.98, SD=1.74, on a scale from 1=”not at all” to 9=”very much”). They also indicated that the negative condition induced negative emotions (*M*=2.92, *SD*=1.54, on a scale from 1=”negative” to 9=”positive”). There were no group differences regarding ‘imagination’ (all *p*’s≥.710, see supplements, Table-S1).

A repeated measures analysis of variance was computed to measure the effectivity of the idiosyncratic emotion induction. PANAS ratings (negative subscale) were entered as dependent variable. Independent variables were the within subject factor Condition (PANAS assessment at: baseline, neutral or negative condition) and the between subject factor Group.

This analysis yielded a significant Condition effect (*F*(2,170)=11.58, *p*<.001, η_p_^2^=.12), with higher negative emotions after the negative condition (*M*=16.95 , *SD*=0.90) than after the neutral condition (*M*=13.68, *SD*=0.59; contrast: *p*<.001) and at baseline (*M*=14.47, *SD*=0.65; contrast: *p*=.005; see supplements Figure-S2A, Table-S4-S5). Further, a significant main effect for Group (*F*(1,85)=5.02, *p*=.028, η_p_^2^=.06) revealed overall higher negative affect in individuals with BED (*M*=16.38, *SD*=1.02) compared to CG (*M*=13.69, *SD*=0.63; see supplements, Figure-S2B and Table-S5). Adding presentation ‘Order’ of negative and neutral condition as between person factor did not change the trend of the Condition effect (see supplements, Table-S6).

*Note*. A) Main effect of Condition; B) Main effect of Group; C) Non-significant interaction Condition and Group. Means on the Negative Positive and Negative Affect Schedule (PANAS) for the ten items assessing negative emotion. Error bars represent the 95% confidence interval around the mean. CG: Control Group; BED: patients with binge-eating disorder. Significance codes: ***=*p*<.001; **=*p*<.010; *=*p*<.050; (*)=*p*<.100.

**Figure-S1**

Group means of negative emotions at baseline and after the neutral and negative condition.


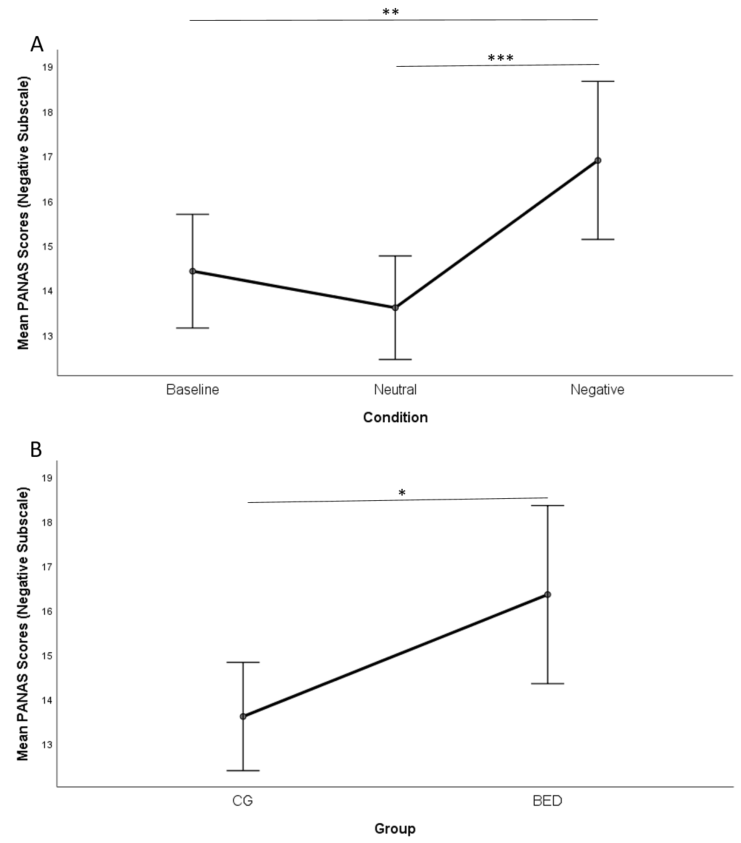

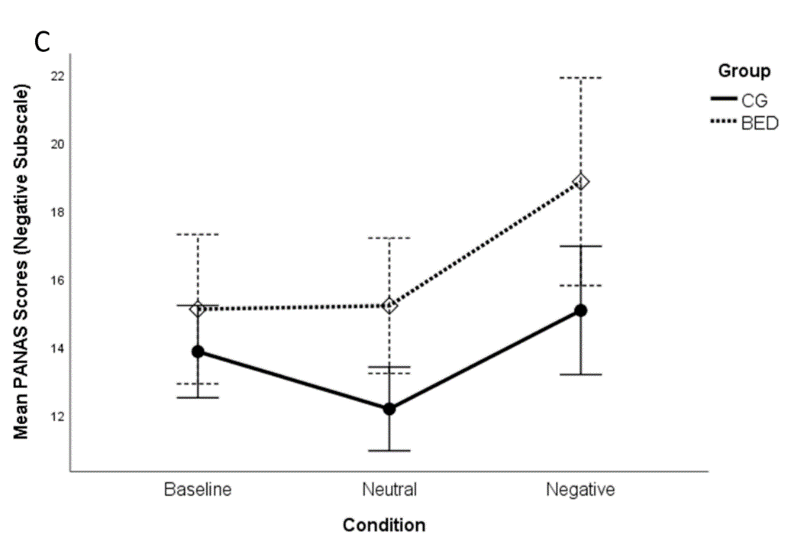


**Table-S4**

Repeated measures analysis of variance for manipulation check of emotion induction.

| Predictor | Sum of Squares | *df* | Mean Square | *F* | *p* | Partial η² |
| --- | --- | --- | --- | --- | --- | --- |
| (Intercept) | **15719.8** | **1** | **15519.8** | **642.6** | **<.001** | **.88** |
| Group | **132.3** | **1** | **132.3** | **5.4** | **.022** | **.06** |
| Condition | **410.9** | **1.5** | **281.9** | **12.1** | **<.001** | **.12** |
| Group * Condition | 65.6 | 1.5 | 44.3 | 1.9 | .165 | .02 |
| Error_within_ | 2961.3 | 126.8 | 23.4 |  |  |  |
| Error_between_ | 2128.2 | 87 | 24.5 |  |  |  |

*Notes.* Due to violated sphericity, p-values and degrees of freedom in the table incorporate the Greenhouse-Geisser correction.

**Table-S5**

Contrasts for the within subject factor Condition of the repeated measures analysis of variance in Table-2.

| Factor Levels | Sum of Squares | | *df* | Mean Square | | | *F* | | | *p* | | | Partial η² | |
| --- | --- | --- | --- | --- | --- | --- | --- | --- | --- | --- | --- | --- | --- | --- |
| Baseline vs. Neutral | 46.5 | | 1 | 46.5 | | 3.2 | | | .077 | | | .04 | |  |
| Baseline vs. Negative | | **428.7** | **1** | **428.7** | **8.6** | | | **.004** | | | **.09** | | |  |
| Neutral vs. Negative | | **757.6** | **1** | **757.6** | **20.1** | | | **<.001** | | | **.19** | | |  |

**Table-S6**

Repeated measures analysis of variance for manipulation check of emotion induction.

| Predictor | Sum of Squares | *df* | Mean Square | *F* | *p* | Partial η² |
| --- | --- | --- | --- | --- | --- | --- |
| (Intercept) | 15590.8 | 1 | 15590.8 | 627.3 | <.001 | .88 |
| Group | **124.9** | **1** | **124.9** | **5.0** | **.028** | **.06** |
| Order | 0.1 | 1 | 0.1 | 0.0 | .959 | .00 |
| Condition | **402.5** | **1.5** | **276.5** | **11.6** | **<.001** | **.12** |
| Group*Order | 11.6 | 1 | 11.6 | 0.5 | .497 | .01 |
| Group*Condition | 58.7 | 1.5 | 40.3 | 1.7 | .195 | .02 |
| Order*Condition | 5.7 | 1.5 | 3.9 | 0.2 | .778 | .00 |
| Group*Condition*Order | 0.6 | 1.5 | 0.4 | 0.0 | .956 | .00 |
| Error_within_ | 2954.3 | 123.7 | 23.9 |  |  |  |
| Error_between_ | 2112.6 | 85 | 24.9 |  |  |  |

*Notes.* Due to violated sphericity, p-values and degrees of freedom in the table incorporate the Greenhouse-Geisser correction. The factor ‘Order’ describes the order in which the negative and neutral condition were presented, as presentation order was randomized between participants.

**Analysis-S2**

**Linear mixed-effects models, post-hoc tests and figures for hypothesis.**

For the analyses of the rating and EMG data, a linear mixed effect modeling (LMM) approach was used to account for variance on the trial level (Nezlek, 2008). First, we tested whether LMMs containing only a random intercept for participant provided a better model fit than general linear regression models. Then, in a stepwise, forward selection process, each fixed factor (Condition, Calories, Group) was modelled as random slope (with the random intercept for participants) and the best fitting model according to the Akaike Information Criterion (AIC) was chosen for each outcome variable (Pleasantness, DTE and EMG).

For the reported models, a random slope for Calories with a random intercept for each participant provided the best model fit (final models: fixed factors were ‘Condition*Calories*Group’ and random effects were a random intercept for participants and a random slope for Calories). Thus, high vs. low Calories had a different influence on the slopes of each participant).

All LMMs were setup with crossed random effect structure (Bliese, 2013) and analyzed in RStudio (Allaire, 2012) using lme4 (Bates et al., 2015) as ratings and EMG activity were nested in participants with the crossed factors Condition and Calories. Contrasts for specific simple slopes of significant interactions were explored with the package lsmeans (Lenth, 2016).

We computed standardized beta coefficients, which allow comparisons of effect sizes between studies (Lorah, 2018) and *conditional pseudo-R²*s (Nakagawa et al., 2017). Still, calculation of effect sizes in LMMs is controversial (Nezlek, 2012). For significant interactions including ‘Group’, post-hoc tests were calculated.

**Table-S7**

Detailed statistics for the reported linear mixed effect models for pleasantness, DTE and Corrugator.

|  | Pleasantness | DTE | Corrugator |
| --- | --- | --- | --- |
| Fixed Effects | **Coefficients β (*SE*)** | **Coefficients β (*SE*)** | **Coefficients β (*SE*)** |
| Intercept | **21.27 (1.81) ***** | **53.67 (2.13) ***** | **-0.05 (0.02) *** |
| Group | 3.49 (3.47) | 6.68 (4.07) | -0.06 (0.05) |
| Condition | **5.00 (1.32) ***** | **6.95 (1.54) ***** | 0.02 (0.03) |
| Calories | -2.52 (2.35) | 0.98 (2.58) | 0.03 (0.03) |
| Group * Condition | **-8.96 (2.48) ***** | **-11.64 (2.87) ***** | **0.12 (0.06) *** |
| Group * Calories | 5.68 (4.50) | 6.97 (4.93) | 0.01 (0.06) |
| Condition * Calories | **-5.25 (1.87) **** | **-5.51 (2.17) *** | -0.04 (0.04) |
| Group * Condition * Calories | 0.25 (3.50) | 2.12 (4.06) | -0.02 (0.08) |
| Random Effects | **Variance (*SD*)** | **Variance (*SD*)** | **Variance (*SD*)** |
| Participant (intercept) | 156.5 (12.51) | 217.7 (14.76) | 0.016 (0.125) |
| Calories (slope) | 245.8 (15.68) | 279.0 (16.70) | 0.007 (0.081) |
| Residual | 739.8 (27.20) | 996.1 (31.56) | 0.554 (0.745) |
| AIC | 45058 | 46469 | 18188 |
| Effect Sizes |  |  |  |
| Marginal pseudo R² | 0.012 | 0.012 | 0.002 |
| Conditional pseudo R² | 0.210 | 0.228 | 0.037 |

*Notes.* Model formula in R for:
Pleasantness model: lmer(Pleasantness ~ Condition * Calories * Group + (Calories|Subjects), data = RATdat);
desire to eat (DTE) model: lmer(DTE ~ Condition * Calories * Group + (Calories|Subjects), data = RATdat);
Corrugator model: lmer(Corrugator ~ Condition * Calories*Group + (Calories|Subjects), data = CORR1dat). Total observations of pleasantness and DTE *N*=4732, Corrugator *N*=8541. Significance codes: ***=*p*<.001; **=*p*<.010; *=*p*<.050; (*)=*p*<.100. Marginal pseudo R² represents the variance explained by the fixed effects, while conditional pseudo R² represents the variance explained by the entire model (including fixed and random effects).

**Table-S8**

Post-hoc tests of all significant two-way interactions of the linear mixed effect models in Table-S2.

| Factors | | Estimate (*SE*) | | *df* | | *p* | |
| --- | --- | --- | --- | --- | --- | --- | --- |
| Pleasantness (Group * Condition) | | | | | | | |
| CG neg vs BED neg | -6.33 (2.93) | | 104 | | .142 | |  |
| CG neg vs. CG neut | **-2.38 (0.94)** | | **4550** | | **.054** | |  |
| CG neg vs. BED neut | 0.12 (2.93) | | 104 | | 1.000 | |  |
| BED neg vs CG neut | 3.96 (2.93) | | 104 | | .534 | |  |
| BED neg vs. BED neut | **6.46 (1.48)** | | **4550** | | **<.001** | |  |
| CG neut vs. BED neut | 2.50 (2.93) | | 104 | | .829 | |  |
| Pleasantness (Calories * Condition) |  | |  | |  | |  |
| lcal neg vs hcal neg | -0.31 (2.25) | | 121 | | .999 | |  |
| lcal neg vs. lcal neut | -0.52 (1.24) | | 4550 | | .975 | |  |
| lcal neg vs. hcal neut | 4.29 (2.25) | | 121 | | .231 | |  |
| hcal neg vs lcal neut | -0.21 (2.25) | | 121 | | 1.000 | |  |
| hcal neg vs. hcal neut | **4.601 (1.24)** | | **4550** | | **.001** | |  |
| lcal neut vs. hcal neut | 4.81 (2.25) | | 121 | | .147 | |  |
| DTE (Group * Condition) | |  | |  | |  | |
| CG neg vs BED neg | **-10.16 (3.74)** | | **101** | | **.038** | |  |
| CG neg vs. CG neut | **-4.19 (1.09)** | | **4550** | | **.001** | |  |
| CG neg vs. BED neut | -3.78 (3.74) | | 101 | | .745 | |  |
| BED neg vs CG neut | 5.97 (3.74) | | 101 | | .386 | |  |
| BED neg vs. BED neut | **6.39 (1.72)** | | **4550** | | **.001** | |  |
| CG neut vs. BED neut | 0.42 (3.74) | | 101 | | 1.000 | |  |
| DTE (Calories * Condition) |  | |  | |  | |  |
| lcal neg vs hcal neg | -4.46 (2.46) | | 126 | | .273 | |  |
| lcal neg vs. lcal neut | -1.13 (1.44) | | 4550 | | .861 | |  |
| lcal neg vs. hcal neut | -1.14 (2.46) | | 126 | | .967 | |  |
| hcal neg vs lcal neut | 3.33 (2.46) | | 126 | | .531 | |  |
| hcal neg vs. hcal neut | **3.32 (1.44)** | | **4550** | | **.095** | |  |
| lcal neut vs. hcal neut | -0.01 (2.46) | | 126 | | 1.000 | |  |
| Corrugator (Group * Condition) | |  | |  | |  | |
| CG neg vs. BED neg | -0.06 (0.05) | | 114 | | .556 | |  |
| CG neut vs. CG neg | 0.00 (0.02) | | 7865 | | .998 | |  |
| BED neut vs. CG neg | -0.05 (0.05) | | 113 | | .644 | |  |
| CG neut vs. BED neg | -0.06 (0.05) | | 114 | | .511 | |  |
| BED neut vs. BED neg | **-0.11 (0.03)** | | **7868** | | **.005** | |  |
| BED neut vs. CG neut | 0.05 (0.05) | | 113 | | .688 | |  |

*Notes.* Post-hoc tests for significat interactions: Control group (CG) and patients with binge-eating disorder (BED); negative (neg) and neutral (neut) Condition; low Calorie Content (lcal) and high Calorie Content (hcal). Desire to eat = DTE. P-values of the contrasts are adjusted for multiple comparisons with Tukey method.


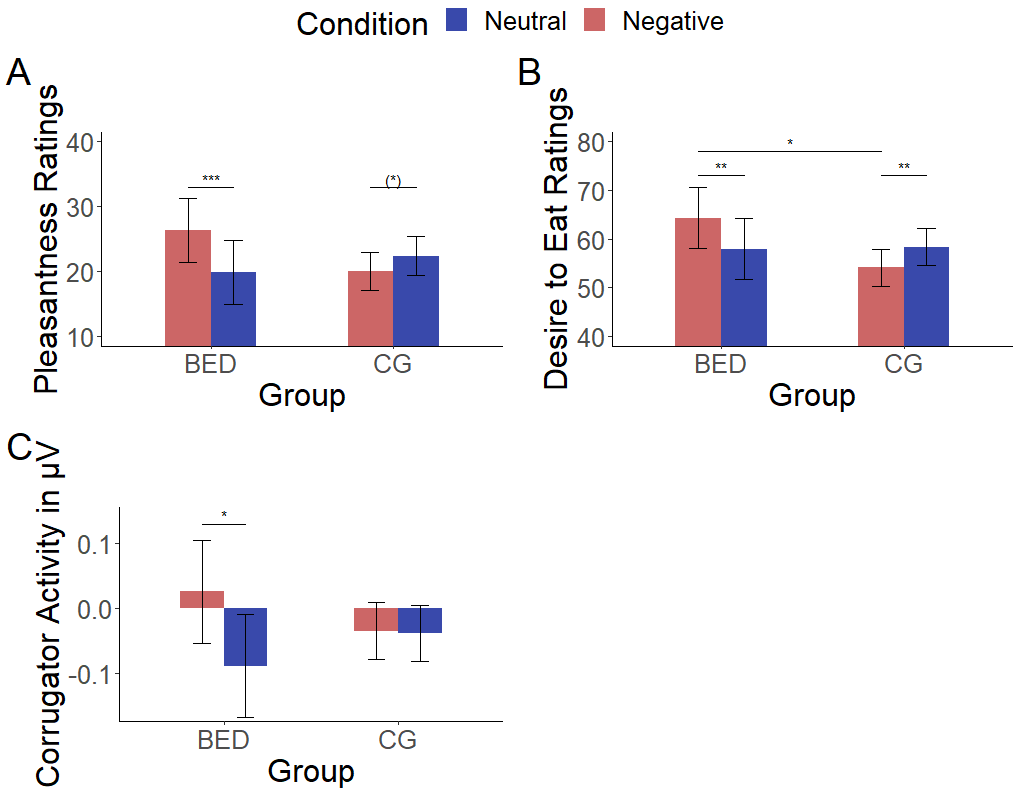


**Figure-2**

Group means in the neutral and negative condition for pleasantness, DTE and Corrugator for food pictures in the neutral and negative condition.

*Note*. (A) Pleasantness ratings of foods – objects difference scores, (B) Desire to eat ratings of food pictures, and (C) Relative Corrugator activity. Corrugator values are baseline corrected, food - object difference scores. Error bars represent the 95% confidence interval around the mean. Control group (CG); patients with binge-eating disorder (BED). (A) & (B): CG *n*=65, BED *n*=24; (C): CG *n*=60, BED *n*=18. See supplementary materials for Figure-S4 & S5, which cover the calorie factor. Significance codes of post-hoc tests for the significant Group * Condition interactions are indicated as: ***=*p*<.001; **=*p*<.010; *=*p*<.050; (*)=*p*<.100.

*Note*. (A) Pleasantness ratings of food - objects difference scores and (B) Desire to eat ratings of food pictures. Error bars represent the 95% confidence interval around the mean. *N* = 89. Significance codes of post-hoc tests for the significant Calorie x Condition interactions are indicated as: ***=*p*<.001; **=*p*<.010; *=*p*<.050; (*)=*p*<.100.

**Figure-S3**

Means of pleasantness and desire to eat for food pictures with low and high calorie content in the neutral and negative condition.


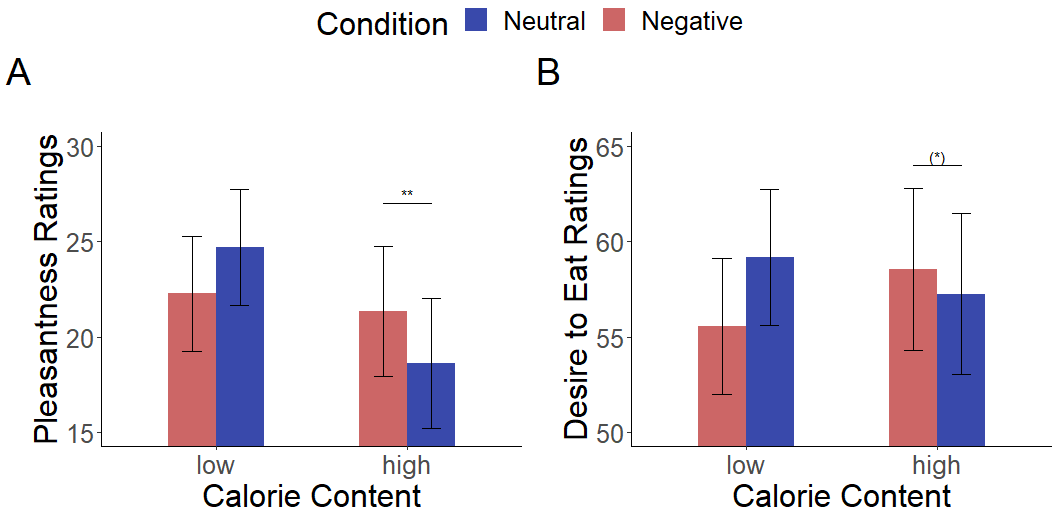


*Note*. Group means of (A) Pleasantness ratings of foods – objects difference scores, (B) Desire to eat ratings of food pictures, and (C) Relative Corrugator activity. Corrugator values are baseline corrected, food - object difference scores. Error bars represent the 95% confidence interval around the mean. CG = control group, BED = patients with binge-eating disorder. (A) & (B): CG *n*=65, BED *n*=24; (C): CG *n*=60, BED *n*=18. As the Group*Condition*Calorie interaction was non-significant no significance codes of post-hoc tests are indicated.

**Figure-S4**

Non-significant higher order three-way-interactions.


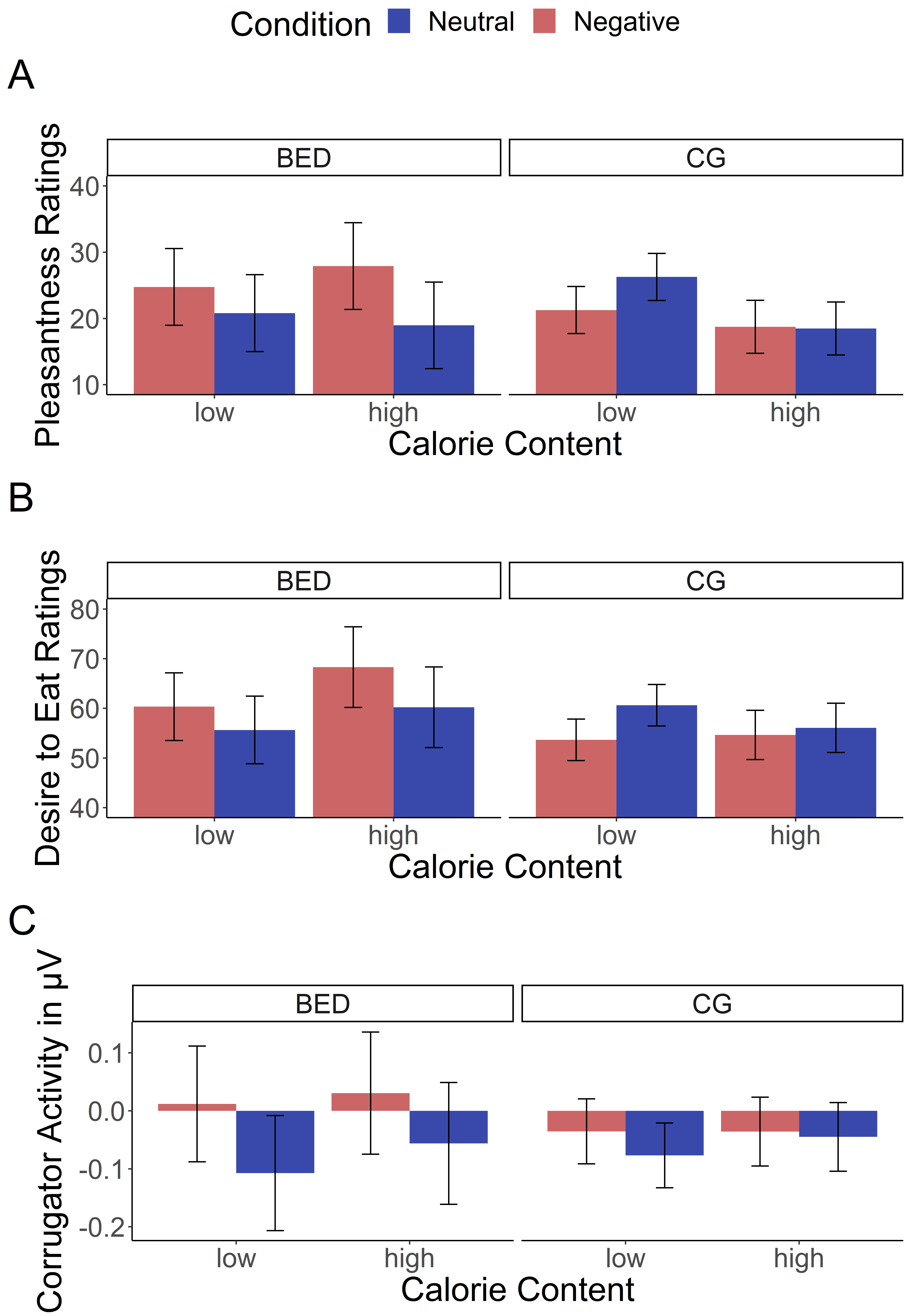


**Linear Mixed-Effects Models with covariates**

**Table-S9**

Detailed statistics for control analysis for study site on pleasantness, DTE and Corrugator.

|  | Pleasantness | DTE | Corrugator |
| --- | --- | --- | --- |
| Fixed Effects | **Coefficients β (*SE*)** | **Coefficients β (*SE*)** | **Coefficients β (*SE*)** |
| Intercept | **20.55 (3.58) ***** | **44.11 (4.32) ***** | -0.05 (0.05) |
| Group | 3.52 (3.60) | 5.00 (4.18) | -0.06 (0.05) |
| Condition | **5.00 (1.32) ***** | **6.95 (1.54) ***** | 0.02 (0.03) |
| Calories | -2.52 (2.35) | 0.98 (2.58) | 0.03 (0.03) |
| Site(SBG) | **0.87 (3.70)** | **11.50 (4.53) *** | -0.01 (0.05) |
| Site(PRI) | **-0.62 (9.17)** | **---** | 0.03 (0.13) |
| Group * Condition | **-8.96 (2.48) ***** | **-11.64 (2.87) ***** | **0.12 (0.06) *** |
| Group * Calories | 5.67 (4.50) | 6.98 (4.93) | 0.01 (0.06) |
| Condition * Calories | **-5.25 (1.87) **** | **-5.51 (2.17) *** | -0.04 (0.04) |
| Group * Condition * Calories | 0.25 (3.50) | 2.12 (4.06) | -0.02 (0.08) |
| Random Effects | **Variance (*SD*)** | **Variance (*SD*)** | **Variance (*SD*)** |
| Participant (intercept) | 159.8 (12.64) | 213.5 (14.61) | 0.016 (0.128) |
| Calories (slope) | 245.8 (15.68) | 279.0 (16.70) | 0.007 (0.084) |
| Residual | 739.8 (27.20) | 996.1 (31.56) | 0.552 (0.743) |
| AIC | 45062 | 46467 | 18192 |
| Effect Sizes |  |  |  |
| Marginal R_GLMM² | 0.012 | 0.024 | 0.002 |
| Conditional R_GLMM² | 0.212 | 0.229 | 0.038 |

*Notes.* Model formula in R for: Pleasantness model: lmer(Pleasantness ~ Condition * Calories * Group + Site + (Calories|Subjects), data = RATdat); desire to eat (DTE) model: lmer(DTE ~ Condition * Calories * Group + Site + (Calories|Subjects), data = RATdat); Corrugator model: lmer(Corrugator ~ Condition * Calories*Group + Site + (Calories|Subjects), data = CORR1dat). Total observations of pleasantness and DTE *N*=4732, Corrugator *N*=8541. Significance codes: ***=*p*<.001; **=*p*<.010; *=*p*<.050; (*)=*p*<.100. Marginal R_GLMM² represents the variance explained by the fixed effects, while conditional R_GLMM² represents the variance explained by the entire model (including fixed and random effects).

**Table-S10**

Detailed statistics for control analysis for BMI on pleasantness, DTE and Corrugator.

|  | | Pleasantness | DTE | Corrugator |
| --- | --- | --- | --- | --- |
| Fixed Effects | | **Coefficients β (*SE*)** | **Coefficients β (*SE*)** | **Coefficients β (*SE*)** |
| Intercept | | **20.98 (1.76) ***** | **53.50 (2.12) ***** | **-0.05 (0.02) *** |
| Group | | 4.64 (3.40) | 7.31 (4.10) . | -0.06 (0.05) |
| Condition | | **5.00 (1.32) ***** | **6.95 (1.54) ***** | 0.02 (0.03) |
| Calories | | -2.52 (2.35) | 0.98 (2.58) | 0.03 (0.03) |
| BMI | **-2.94 (1.22) *** | -1.61 (1.59) | 0.00 (0.02) | |
| Group * Condition | | **-8.96 (2.48) ***** | **-11.64 (2.87) ***** | **0.12 (0.06) *** |
| Group * Calories | | 5.68 (4.50) | 6.97 (4.93) | 0.01 (0.06) |
| Condition * Calories | | **-5.25 (1.87) **** | **-5.51 (2.17) *** | -0.04 (0.04) |
| Group * Condition * Calories | | 0.25 (3.50) | 2.12 (4.06) | -0.02 (0.08) |
| Random Effects | | **Variance (*SD*)** | **Variance (*SD*)** | **Variance (*SD*)** |
| Participant (intercept) | | 144.5 (12.02) | 214.8 (14.66) | 0.016 (0.126) |
| Calories (slope) | | 245.8 (15.68) | 278.9 (16.70) | 0.007 (0.081) |
| Residual | | 739.8 (27.20) | 996.1 (31.56) | 0.554 (0.745) |
| AIC | | 45054 | 46470 | 18190 |
| Effect Sizes | |  |  |  |
| Marginal R_GLMM² | | 0.021 | 0.014 | 0.002 |
| Conditional R_GLMM² | | 0.212 | 0.231 | 0.038 |

*Notes.* Model formula in R for: Pleasantness model: lmer(Pleasantness ~ Condition * Calories * Group + BMI_gmc + (Calories|Subjects), data = RATdat); desire to eat (DTE) model: lmer(DTE ~ Condition * Calories * Group + BMI_gmc + (Calories|Subjects), data = RATdat); Corrugator model: lmer(Corrugator ~ Condition * Calories * Group + BMI_gmc + (Calories|Subjects), data = CORR1dat). Total observations of pleasantness and DTE *N*=4732, Corrugator *N*=8541. Significance codes: ***=*p*<.001; **=*p*<.010; *=*p*<0.05; (*)=*p*<.100. Marginal R_GLMM² represents the variance explained by the fixed effects, while conditional R_GLMM² represents the variance explained by the entire model (including fixed and random effects).

**Table-S11**

Detailed statistics for control analysis for ‘compliance with standardized lunch options pre laboratory session’ on pleasantness, DTE and Corrugator.

|  | Pleasantness | DTE | Corrugator |
| --- | --- | --- | --- |
| Fixed Effects | **Coefficients β (*SE*)** | **Coefficients β (*SE*)** | **Coefficients β (*SE*)** |
| Intercept | **21.38 (1.83) ***** | **53.64 (2.15) ***** | **-0.05 (0.02) *** |
| Group | 4.12 (3.56) | 7.06 (4.20) (*) | -0.05 (0.05) |
| Condition | **5.00 (1.32) ***** | **6.95 (1.54) ***** | 0.02 (0.03) |
| Calories | -2.52 (2.35) | 0.98 (2.58) | 0.03 (0.03) |
| Non-compliant (ate more) | -7.11 (6.96) | 1.94 (8.86) | -0.11 (0.09) |
| Non-compliant (ate less) | -3.55 (11.95) | -12.56 (15.19) | 0.08 (0.16) |
| Group * Condition | **-8.96 (2.48) ***** | **-11.64 (2.87) ***** | **0.12 (0.06) *** |
| Group * Calories | 5.68 (4.50) | 6.97 (4.93) | 0.01 (0.06) |
| Condition * Calories | **-5.25 (1.87) **** | **-5.51 (2.17) *** | -0.04 (0.04) |
| Group * Condition * Calories | 0.25 (3.50) | 2.12 (4.06) | -0.02 (0.08) |
| Random Effects | **Variance (*SD*)** | **Variance (*SD*)** | **Variance (*SD*)** |
| Participant (intercept) | 159.4 (12.63) | 221.6 (14.89) | 0.016 (0.125) |
| Calories (slope) | 245.8 (15.68) | 279.0 (16.70) | 0.007 (0.081) |
| Residual | 739.8 (27.20) | 996.1 (31.56) | 0.554 (0.745) |
| AIC | 45061 | 46472 | 18191 |
| Effect Sizes |  |  |  |
| Marginal R_GLMM² | 0.014 | 0.014 | 0.003 |
| Conditional R_GLMM² | 0.212 | 0.231 | 0.038 |

*Notes.* Model formula in R for: Pleasantness model: lmer(Pleasantness ~ Condition * Calories * Group + Compliance + (Calories|Subjects), data = RATdat); desire to eat (DTE) model: lmer(DTE ~ Condition * Calories * Group + Compliance + (Calories|Subjects), data = RATdat); Corrugator model: lmer(Corrugator ~ Condition * Calories*Group + Compliance + (Calories|Subjects), data = CORR1dat). Total observations of pleasantness and DTE *N*=4732, Corrugator *N*=8541. Significance codes: ***=*p*<.001; **=*p*<.010; *=*p*<.050; (*)=*p*<.100. Marginal R_GLMM² represents the variance explained by the fixed effects, while conditional R_GLMM² represents the variance explained by the entire model (including fixed and random effects).

**Analysis-S3**

**A priori and post-hoc sample size and power simulations.**

We used the R package ‘simr’ (Green et al., 2016) on a data set of our previous study (Schnepper et al., 2021) with an identical task and similar analyses in anorexia nervosa (AN), bulimia nervosa (BN) and matched controls to conduct an a priori power analysis. The a priori power simulation reveled that a sample size of approximately *N*=360 would be needed to replicate the results of that study regarding findings in BN (i.e., 3-way interactions for DTE and EMG ‘Group*Condition*Calories’; see supplement, Table-S12 and S13).

However, due to recruitment difficulties, we were unable to acquire the aspired sample size. Thus, we additionally conducted a post-hoc power analysis on the present data. The post-hoc power simulation reveled that higher *N*’s (especially in the small BED sample) would have been needed to obtain statistical power at the recommended .80 level to rule out the possibility that the hypothesized three-way interactions remained undetected (Cohen, 1988; see supplement, Table-S14).

Thus, limited statistical power due to the small sample size of individuals with BED (*n*=24/18) may have limited the detection of findings for some of the statistical comparisons conducted (i.e., the three-way interactions ‘Group*Condition*Calories’ and all effects reading the dependent variable EMG). In addition, we only found small effect sizes for the EMG LMM. Thus, this study provides only preliminary evidence regarding the results on EMG (i.e., emotion potentiation of aversive-defensive reactivity in BED) and three-way interactions.

Still, the sample size was sufficient to reach acceptable power levels (.75-.97) for the reported 2-way interactions (‘Group*Condition’ and ‘Condition*Calories’) regarding the dependent variables pleasantness and DTE. Thus, the results still strongly support emotion potentiated food-cue reactivity in BED – at least with regard to appetitive ratings.

**Table-S12**

Results in Schnepper et al. (2021), which were used for a priori power analyses.

| Factors | Pleasantness | Desire to eat | Corrugator |
| --- | --- | --- | --- |
| Fixed effects | **Coefficients β (*SE*)** | **Coefficients β (*SE*)** | **Coefficients β (*SE*)** |
| (Intercept) | **69.8 (2.03)***** | **55.0 (2.59)***** | −0.08 (0.06) |
| Condition | **7.79 (1.77)***** | **7.22 (2.33)**** | 0.03 (0.06) |
| Calories | −1.83 (3.06) | −0.78 (3.03) | 0.02 (0.06) |
| Group (AN) | **−8.19 (3.34)*** | **−20.66 (4.26)***** | **0.23 (0.10)*** |
| Group (BN) | **−4.19 (3.38)** | **−4.57 (4.32)** | **0.17 (0.11)^(^*^)^** |
| Condition * Calories | −3.48 (2.50) | −4.43 (3.30) | 0.01 (0.08) |
| Condition * AN | −0.48 (2.91) | −0.10 (3.84) | −0.03 (0.10) |
| Condition * BN | **−5.49 (2.96)^(^*^)^** | **−9.88 (3.90)*** | **−0.34 (0.10)***** |
| Calories * AN | **−30.54 (5.04)***** | **−10.9 (5.00)*** | −0.11 (0.11) |
| Calories * BN | −2.72 (5.12) | **14.9 (5.08)**** | −0.21 (0.11)^(^*^)^ |
| Condition * Calories * AN | 1.60 (4.12) | −0.46 (5.43) | −0.04 (0.14) |
| Condition * Calories * BN | −5.59 (4.19) | **−14.27 (5.51)*** | **0.31 (0.14)*** |
| Random effects | **Variance (*SD*)** | **Variance (*SD*)** | **Variance (*SD*)** |
| Participant (intercept) | 178 (13.3) | 278 (16.7) | 0.13 (0.36) |
| Calories (slope) | 438 (20.9) | 265 (16.3) | 0.06 (0.24) |
| Residual | 110 (10.5) | 190 (13.8) | 0.11 (0.33) |
| AIC | 5055 | 5277 | 538 |

*Note*. Coefficients of effects in BN (patients with bulimia nervosa) were used to predict sample sizes in the current study. Anorexia nervos (AN, *n*=41), BN (*n*=39), and control group (*n*=70). Significance codes: ***=*p*<.001; **=*p*<.010; *=*p*<.050; (*)=*p*<.100.

**Table-S13**

A priori sample size and power simulations based on data from Schnepper et al. (2021) for fixed and random effects as found in the current papers linear mixed effect models in Table-S2.

| Simulated Power Level (1-*β* err prob) for Sample Size *N* | | | | | | | |
| --- | --- | --- | --- | --- | --- | --- | --- |
| Fixed Effects of Model X | ***N*=30** | ***N*=60** | ***N*=90** | ***N*=120** | ***N*=150** | ***N*=180 […]** | ***N=*360** |
| Pleasantness |  |  |  |  |  |  |  |
| Condition | 0.52 | 0.79 | 0.92 | 0.98 | 1.00 | 0.89 | 0.97 |
| Calories | 0.01 | 0.00 | 0.02 | 0.02 | 0.03 | 0.07 | 0.11 |
| Group AN | 0.15 | 0.31 | 0.53 | 0.73 | 0.83 | 0.77 | 0.95 |
| Group BN | 0.03 | 0.04 | 0.07 | 0.13 | 0.20 | 0.25 | 0.41 |
| Condition*Calories | 0.10 | 0.17 | 0.22 | 0.24 | 0.24 | 0.14 | 0.27 |
| Condition*Group AN | 0.06 | 0.04 | 0.08 | 0.04 | 0.07 | 0.06 | 0.07 |
| Condition*Group BN | 0.11 | 0.23 | 0.31 | 0.39 | 0.45 | 0.29 | 0.38 |
| Calories*Group AN | 0.88 | 1.00 | 1.00 | 1.00 | 1.00 | 1.00 | 1.00 |
| Calories*Group BN | 0.01 | 0.00 | 0.01 | 0.02 | 0.01 | 0.04 | 0.10 |
| Condition*Calories*Group AN | 0.05 | 0.08 | 0.07 | 0.05 | 0.08 | 0.05 | 0.08 |
| Condition*Calories*Group BN | 0.08 | 0.08 | 0.14 | 0.19 | 0.21 | 0.13 | 0.21 |
| DTE |  |  |  |  |  |  |  |
| Condition | 0.23 | 0.52 | 0.67 | 0.73 | 0.83 | 0.68 | 0.89 |
| Calories | 0.03 | 0.04 | 0.02 | 0.01 | 0.01 | 0.04 | 0.05 |
| Group AN | 0.74 | 0.74 | 1.00 | 1.00 | 1.00 | 1.00 | 1.00 |
| Group BN | 0.04 | 0.04 | 0.05 | 0.08 | 0.09 | 0.17 | 0.37 |
| Condition*Calories | 0.11 | 0.11 | 0.18 | 0.20 | 0.28 | 0.17 | 0.24 |
| Condition*Group AN | 0.04 | 0.04 | 0.04 | 0.07 | 0.05 | 0.06 | 0.05 |
| Condition*Group BN | 0.23 | 0.23 | 0.57 | 0.58 | 0.69 | 0.50 | 0.74 |
| Calories*Group AN | 0.12 | 0.12 | 0.43 | 0.64 | 0.68 | 0.61 | 0.71 |
| Calories*Group BN | 0.26 | 0.26 | 0.72 | 0.84 | 0.95 | 0.84 | 0.96 |
| Condition*Calories*Group AN | 0.04 | 0.04 | 0.04 | 0.05 | 0.07 | 0.03 | 0.05 |
| Condition*Calories*Group BN | 0.26 | 0.26 | 0.58 | 0.66 | 0.75 | 0.56 | 0.80 |
| Corrugator |  |  |  |  |  |  |  |
| Condition | 0.04 | 0.04 | 0.08 | 0.06 | 0.08 | 0.10 | 0.11 |
| Calories | 0.04 | 0.03 | 0.03 | 0.02 | 0.03 | 0.05 | 0.05 |
| Group AN | 0.14 | 0.35 | 0.50 | 0.67 | 0.73 | 0.87 | 0.98 |
| Group BN | 0.06 | 0.14 | 0.24 | 0.24 | 0.37 | 0.48 | 0.79 |
| Condition*Calories | 0.08 | 0.04 | 0.05 | 0.03 | 0.05 | 0.08 | 0.04 |
| Condition*Group AN | 0.07 | 0.04 | 0.03 | 0.03 | 0.08 | 0.06 | 0.07 |
| Condition*Group BN | 0.33 | 0.61 | 0.80 | 0.89 | 0.85 | 0.92 | 1.00 |
| Calories*Group AN | 0.06 | 0.06 | 0.10 | 0.16 | 0.17 | 0.15 | 0.27 |
| Calories*Group BN | 0.08 | 0.26 | 0.36 | 0.44 | 0.44 | 0.51 | 0.73 |
| Condition*Calories*Group AN | 0.08 | 0.03 | 0.07 | 0.04 | 0.08 | 0.07 | 0.05 |
| Condition*Calories*Group BN | 0.17 | 0.32 | 0.48 | 0.51 | 0.55 | 0.57 | 0.81 |

*Notes.* Desire to eat (DTE). Significance codes: ***=*p*<.001; **=*p*<.010; *=*p*<.050; (*)=*p*<.100. Dataset included three groups: anorexia nervosa (AN), bulimia nervosa (BN) and a healthy control group (matched to BN in body-mass index and age). For effects see Table-S12. Actual sample sizes: AN *n*=41, BN *n*=39, and control group *n*=70.

**Table-S14**

Post-hoc sample sizes and power simulation for fixed effects as found in the pleasantness, DTE and corrugator linear mixed effect models in Table-S2 based on the current data.

|  | Simulated Power Level (1-*β* err prob) for Sample Size *N* | | | | | |
| --- | --- | --- | --- | --- | --- | --- |
| Fixed Effects of Model X | *N*=36 | *N*=48 | *N*=78 | *N*=89 | *N*=100 | *N*=120 |
| Pleasantness |  |  |  |  |  |  |
| Group |  | 0.04 |  | 0.17 | 0.24 | 0.32 |
| Condition |  | 0.32 |  | 0.45 | 0.46 | 0.58 |
| Calories |  | 0.05 |  | 0.13 | 0.26 | 0.23 |
| Group x Condition |  | 0.74 |  | 0.95 | 0.96 | 0.98 |
| Group x Calories |  | 0.06 |  | 0.14 | 0.14 | 0.20 |
| Condition x Calories |  | 0.49 |  | 0.79 | 0.79 | 0.80 |
| Group x Condition x Calories |  | 0.03 |  | 0.04 | 0.04 | 0.04 |
| DTE |  |  |  |  |  |  |
| Group |  | 0.14 |  | 0.35 | 0.40 | 0.51 |
| Condition |  | 0.10 |  | 0.11 | 0.09 | 0.09 |
| Calories |  | 0.01 |  | 0.02 | 0.04 | 0.04 |
| Group x Condition |  | 0.84 |  | 0.97 | 0.99 | 1.00 |
| Group x Calories |  | 0.11 |  | 0.23 | 0.22 | 0.35 |
| Condition x Calories |  | 0.50 |  | 0.75 | 0.76 | 0.82 |
| Group x Condition x Calories |  | 0.08 |  | 0.06 | 0.09 | 0.10 |
| Corrugator |  |  |  |  |  |  |
| Group | 0.01 |  | 0.02 |  | 0.05 | 0.04 |
| Condition | 0.10 |  | 0.20 |  | 0.21 | 0.26 |
| Calories | 0.05 |  | 0.04 |  | 0.04 | 0.02 |
| Group x Condition | 0.29 |  | 0.52 |  | 0.70 | 0.76 |
| Group x Calories | 0.07 |  | 0.02 |  | 0.05 | 0.02 |
| Condition x Calories | 0.12 |  | 0.19 |  | 0.21 | 0.20 |
| Group x Condition x Calories | 0.06 |  | 0.05 |  | 0.04 | 0.05 |

*Notes.* Model formula in R for:
Pleasantness model: lmer(Pleasantness ~ Condition * Calories * Group + (Calories|Subjects), data = RATdat);
desire to eat (DTE) model: lmer(DTE ~ Condition * Calories * Group + (Calories|Subjects), data = RATdat);
Corrugator model: lmer(Corrugator ~ Condition * Calories*Group + (Calories|Subjects), data = CORR1dat). . Significance codes: ***=*p*<.001; **=*p*<.010; *=*p*<.050; (*)=*p*<.100. Pleasantness & DTE models: CG *n*=65, BED *n*=24, *N*=89; Corrugator model: CG *n*=60, BED *n*=18, *N*=78.

**Other materials**

**Table-S15**

Papers focusing on other parts of the ‘NewEat’ project.

|  | DOI |
| --- | --- |
| Unrelated Topics |  |
| Emotion regulation in AN and BN | 10.1080/10640266.2019.1642036 |
| Review on emotional eating | 10.1017/S0029665120007004 |
| Interoceptive sensitivity and intuitive eating in AN and BN | 10.1002/erv.2676 |
| Decision-making in healthy individuals | 10.1007/s00426-019-01185-3 |
| Decision-making in AN, BN and matched controls | 10.1016/j.appet.2021.105745 |
| Decision-making in BED and matched controls | 10.1016/j.appet.2021.105890 |
| Related Topics |  |
| Emotion potentiated food-cue reactivity and brain activity in healthy individuals | 10.3389/fnbeh.2020.00091 |
| Emotion potentiated food-cue reactivity and brain activity in AN, BN and matched controls | [10.1002/erv.2849](https://doi.org/10.1002/erv.2849) |
| Emotion related brain activity in BN and matched controls | [10.1016/j.addbeh.2020.106712](https://doi.org/10.1016/j.addbeh.2020.106712) |

*Notes.* The paper regarding food cue responses and brain activity under negative emotions use parts of the control group data used in this manuscript. Anorexia nervosa (AN), bulimia nervosa (BN), binge-eating Disorder (BED)

**Table-S16**

Included food and object picture stimuli with their numbers in the food-pics_extended database (Blechert et. al. 2019; <https://doi.org/10.3389/fpsyg.2019.00307>)

| **High-caloric food** | | **Low-caloric food** | | **Objects** | |
| --- | --- | --- | --- | --- | --- |
| **picture name** | **picture** | **picture name** | **picture** | **picture name** | **picture** |
| 0022.jpg | **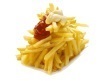** | 0192.jpg | 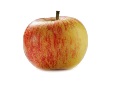 | 1005.jpg | 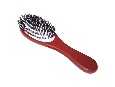 |
| 0061.jpg | **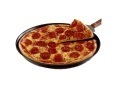** | 0197.jpg | 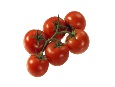 | 1008.jpg | 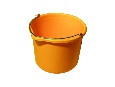 |
| 0065.jpg | 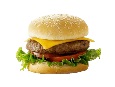 | 0198.jpg | 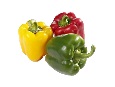 | 1015.jpg | 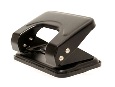 |
| 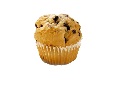0080.jpg |  | 0202.jpg | 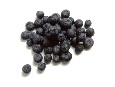 | 1019.jpg | 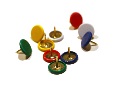 |
| 0117.jpg | 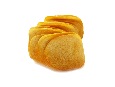 | 0225.jpg | 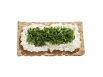 | 1028.jpg | 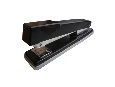 |
| 0153.jpg | 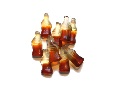 | 0228.jpg | 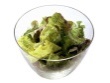 | 1094.jpg | 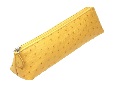 |
| 0161.jpg | 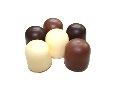 | 0230.jpg | 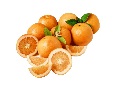 | 1113.jpg | 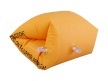 |
| 0183.jpg | 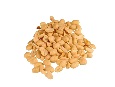 | 0263.jpg | 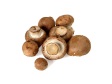 | 1129.jpg | 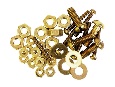 |
| 0184.jpg | 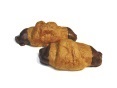 | 0268.jpg | 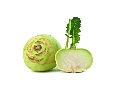 | 1133.jpg | 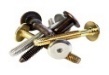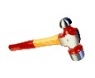 |
| 0286.jpg | 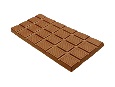 | 0334.jpg | 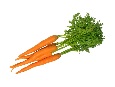 | 1139.jpg |  |
| 0309.jpg | 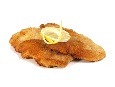 | 0379.jpg | 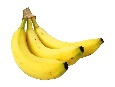 | 1140.jpg | 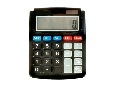 |
| 0519.jpg | 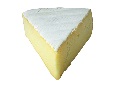 | 0380.jpg | 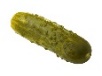 | 1144.jpg | 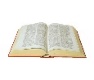 |
| 0535.jpg | 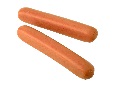 | 0454.jpg | 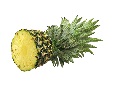 | 1147.jpg | 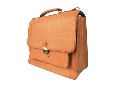 |
|  |  |  |  | 1154.jpg | 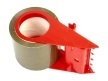 |
|  |  |  |  | 1155.jpg | 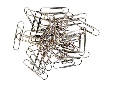 |
|  |  |  |  | 1200.jpg | 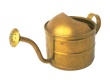 |
|  |  |  |  | 1211.jpg | 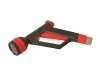 |
|  |  |  |  | 1213.jpg | 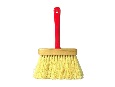 |
|  |  |  |  | 1214.jpg | 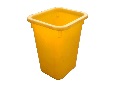 |
|  |  |  |  | 1218.jpg | 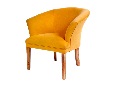 |
|  |  |  |  | 1241.jpg | 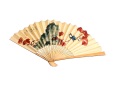 |
|  |  |  |  | 1250.jpg | 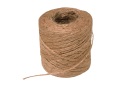 |
|  |  |  |  | 1256.jpg | 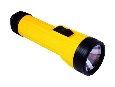 |
|  |  |  |  | 1266.jpg | 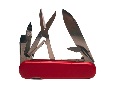 |
|  |  |  |  | 1268.jpg | 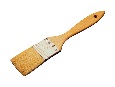 |
|  |  |  |  | 1279.jpg | 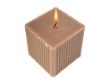 |

**References (Supplement)**

Allaire, J. (2012). RStudio: integrated development environment for R. *Boston, MA*.

Bates, D., Maechler, M., Bolker, B., & Walker, S. (2015). Fitting Linear Mixed-Effects Models Using lme4. *Journal of Statistical Software, 67*(1), 1-48. https://doi.org/10.18637/jss.v067.i01

Bliese, P. D. (2013). *Multilevel modeling in R (2.5)*. Retrieved September 3 from

Green, P., MacLeod, C. J., & Alday, P. (2016). simr: an R package for power analysis of generalised linear mixed models by simulation. *Methods in Ecology and Evolution, 7*, 493-498.

Grimm, J. W. (2009). State-trait-anxiety inventory nach spielberger. deutsche lang-und kurzversion.

Hilbert, A., & Tuschen-Caffier, B. (2006). *Eating Disorder Examination - Questionnaire: Deutschsprachige Übersetzung*. Verlag für Psychotherapie.

Hilbert, A., Tuschen-Caffier, B., & Ohms, M. (2004). Eating Disorder Examination: Deutschsprachige Version des strukturierten Essstörungsinterviews. *Diagnostica, 50*, 98-106.

Lenth, R. (2016). *Least-squares means: the R package lsmeans. J Stat Sofw 69: 1–33.* In

Lorah, J. (2018). Effect size measures for multilevel models: Definition, interpretation, and TIMSS example. *Large-Scale Assessments in Education, 6*(1), 8.

Meule, A., Reichenberger, J., & Blechert, J. (2018). Development and Preliminary Validation of the Salzburg Emotional Eating Scale. *Front Psychol, 9*, 88. https://doi.org/10.3389/fpsyg.2018.00088

Meule, A., Vögele, C., & Kübler, A. (2011). Psychometric evaluation of the German Barratt Impulsiveness Scale - Short Version (BIS-15). *Diagnostica, 57*, 126-133.

Nakagawa, S., Johnson, P. C. D., & Schielzeth, H. (2017). The coefficient of determination *R*² and intra-class correlation coefficient from generalized linear mixed-effects models revisited and expanded. *Journal of The Royal Society Interface, 14*(134), 20170213. https://doi.org/10.1098/rsif.2017.0213

Nezlek, J. B. (2008). An introduction to multilevel modeling for social and personality psychology. *Social and Personality Psychology Compass, 2*(2), 842-860.

Nezlek, J. B. (2012). Multilevel modeling for psychologists. In *APA handbook of research methods in psychology, Vol 3: Data analysis and research publication.* (pp. 219-241). American Psychological Association. https://doi.org/10.1037/13621-011

Paul, T., & Thiel, A. (2005). *EDI-2: Eating Disorder Inventory-2 - Deutsche Version - Manual*. Hogrefe.

Radloff, L. S. (1977). The CES-D scale: A self-report depression scale for research in the general population. *Applied Psychological Measurement, 1*, 385-401.

Schnepper, R., Richard, A., Georgii, C., Arend, A.-K., Naab, S., Voderholzer, U., Wilhelm, F. H., & Blechert, J. (2021). Bad mood food? Increased versus decreased food cue reactivity in Anorexia Nervosa and Bulimia Nervosa during negative emotions. *European Eating Disorders Review*.

Spielberger, C. D. (1983). *Manual for the State-Trait Anxiety Inventory STAI (Form Y)*. Consulting Psychologists Press.

van Strien, T., Frijters, J. E., Bergers, G., & Defares, P. B. (1986). The Dutch Eating Behavior Questionnaire (DEBQ) for assessment of restrained, emotional, and external eating behavior. *International Journal of Eating Disorders, 5*(2), 295-315. https://doi.org/10.1002/1098-108X(198602)5:2<295::AID-EAT2260050209>3.0.CO;2-T

Wittchen, H., Zaudig, M., & Fydrich, T. (1997). *Strukturiertes Klinisches Interview für DSM-IV. Achse I und II. Handanweisung.* Hogrefe.
